# Pan-cancer analysis of SYNGR2 with a focus on clinical implications and immune landscape in liver hepatocellular carcinoma

**DOI:** 10.1186/s12859-023-05323-y

**Published:** 2023-05-11

**Authors:** Chunxun Liu, Zhaowei Qu, Haoran Zhao, Peng Wang, Chao Zhan, Yubao Zhang

**Affiliations:** Department of Hepatopancreatobiliary Surgery, Harbin Medical University Cancer Hospital, Harbin, China

**Keywords:** SYNGR2, Pan-cancer, LIHC, Prognosis, Immune analysis

## Abstract

**Background:**

Synaptogyrin-2 (SYNGR2), as a member of synaptogyrin gene family, is overexpressed in several types of cancer. However, the role of SYNGR2 in pan-cancer is largely unexplored.

**Methods:**

From the TCGA and GEO databases, we obtained bulk transcriptomes, and clinical information. We examined the expression patterns, prognostic values, and diagnostic value of SYNGR2 in pan-cancer, and investigated the relationship of SYNGR2 expression with tumor mutation burden (TMB), microsatellite instability (MSI), immune infiltration, and immune checkpoint (ICP) genes. The gene set enrichment analysis (GSEA) software was used to perform pathway analysis. Besides, we built a nomogram of liver hepatocellular carcinoma patients (LIHC) and validated its prediction accuracy.

**Results:**

SYNGR2 was highly expressed in most cancers. The high expression of SYNGR2 significantly reduced the overall survival (OS), disease-specific survival (DSS), disease-free interval (DFI), and progression-free interval (PFI) in multiple types of cancer. Also, receiver operating characteristic (ROC) curve analysis demonstrated that SYNGR2 showed high accuracy in distinguishing cancerous tissues from normal ones. Moreover, SYNGR2 expression was correlated with TMB, MSI, immune scores, and immune cell infiltrations. We also analyzed the association of SYNGR2 with immunotherapy response in LIHC. Finally, a nomogram including SYNGR2 and pathologic T, N, M stage was built and exhibited good predictive power for the OS, DSS, and PFI of LIHC patients.

**Conclusion:**

Overall, SYNGR2 is a critical oncogene in various tumors. SYNGR2 participates in the carcinogenic progression, and may contribute to the immune infiltration in tumor microenvironment. Our study suggests that SYNGR2 can serve as a predictor related to prognosis in pan-cancer, especially LIHC.

**Supplementary Information:**

The online version contains supplementary material available at 10.1186/s12859-023-05323-y.

## Introduction

Cancer is a major disease that seriously threatens human health and life worldwide, and the incidence and mortality are rapidly increasing globally [1]. The treatment of cancers has always been a pressing challenge for the medical community. Cancer patients have experienced significant improvements thanks to advances in surgery, molecular-targeted therapy and immunotherapy in recent years. However, the 5-year survival rate of cancer patients after diagnosis remains discouraging [2]. Consequently, there is a dire need to clarify the molecular mechanisms elucidating patterns of cancer pathogenesis and to identify reliable biomarkers for the early detection, diagnosis and treatment of cancers [3].

Synaptogyrin-2 (SYNGR2), also known as Cellugyrin, belongs to the synaptogyrin gene family. SYNGR2 gene is located in chromosome 17q25.3, encoding a protein product composed of 224 amino acids [4]. Previous studies have found that SYNGR2 plays an important role in cellular exocytosis, the storage and transport of GLUT4 at the cytoplasmic membrane, and the formation and maturation of microvesicles in neuronal cells [5]. At present, the studies on synaptogyrin gene family are still in their infancy. SYNGR3 was found to be expressed in the cytoplasm of chromophobe renal cell carcinoma, but not in the cytoplasm of renal oncocytoma. Accordingly, SYNGR3 may be used as the basis for the diagnosis of renal cell carcinoma in the future [6]. With the continuous progress of research, Bin Li et al. [7] found that SYNGR2 was associated with poorer overall survival, poorer disease-specific survival and T stage in ESCC. SYNGR2 may be used as a biomarker for determining prognosis and immune infiltration in ESCC. However, the effects of enhanced SYNGR2 gene expression on prognosis have not been systematically evaluated across different cancer types. In the last twenty years, comprehensive genomic characterization of tumors has become a major goal in the field of cancer research. The development of molecular histology and next-generation sequencing technologies has dramatically changed the study of cancer [8]. Large-scale genomics projects provide matched molecular and clinical data of various cancers, which helps systematically analyze the survival impact of single gene expression. Therefore, this is advantageous for analyzing and revealing potential biomarkers’ prognostic values with Pan-cancer analysis.

In this study, we examined the expression of SYNGR2 across 33 cancer types. Meanwhile, we also evaluated the prognostic value of SYNGR2 in pan-cancer based on multiple databases. Moreover, this study totally explored the potential association of SYNGR2 with clinical characteristics, tumor mutation burden (TMB), microsatellite instability (MSI), immune infiltration, and immune checkpoint genes. Finally, we found that the expression of SYNGR2 was significantly associated with survival prognosis in liver hepatocellular carcinoma (LIHC), and had a high diagnostic value in LIHC. Therefore, we constructed a SYNGR2-related prognostic risk-score model for LIHC patients and identified signal pathways that SYNGR2 regulates the development of LIHC. The current study indicates that SYNGR2 has promise as a prognostic biomarker in various cancers. These findings may have important implications in guiding basic research as well as clinical practice.

## Materials and methods

### Data collection

The RNA sequencing and clinical information for 33 types of cancers were acquired from the Cancer Genome Atlas (TCGA, https://portal.gdc.cancer.gov/), and the Genotype-Tissue Expression (GTEx, https://www.genome.gov/Funded-Programs-Projects/Genotype-Tissue-Expression-Project). The Single‑cell data of SYNGR2 was obtained from the Tabula Muris (https://tabula-muris.ds.czbiohub.org/). To verify the predictive value of SYNGR2 in LIHC, gene expression data and clinical data of GSE14520 were downloaded from the GEO (https://www.ncbi.nlm.nih.gov/geo) database. First, the expression levels of SYNGR2 in normal and tumor tissues were compared using the Wilcoxon rank sum test function in the “ggplot2” R package. Additionally, an online tool, the Gene Expression Profiling Interactive Analysis (GEPIA) (http://gepia.cancer-pku.cn/), was adopted to visualize the SYNGR2 expression levels in different stages of all tumors. GEPIA comprised gene expression information from TCGA and gene expression profiles for normal tissues from the GTEx database [9]. Finally, we explored the protein expression level of SYNGR2 between primary tumors and normal tissues through the UALCAN portal (http://ualcan.path.uab.edu/analysis-prot.html) [10, 11].

### Analysis of survival and prognosis

The pan-cancer samples were divided into SYNGR2^high^ and SYNGR2^low^ expression groups based on the minimum *p*-value approach. The Kaplan–Meier survival curves were utilized to exhibit the correlation of SYNGR2 expression with the prognosis of patients’ overall survival (OS), disease-specific survival (DSS), progression-free interval (PFI), and disease-free interval (DFI). We also implemented univariate Cox regression analysis to estimate the prognostic value of SYNGR2 by calculating the hazard ratio (HR) and 95% confidence interval (CI) by executing the “survival” and “forestplot” R packages.

### SYNGR2’s capacity to distinguish tumor from non-tumor tissues

A ROC analysis of SYNGR2 expression levels was performed using the “pROC” R package to examine whether SYNGR2 expression levels can separate tumors and normal tissue across the 33 types of cancer, and the area under curve (AUC) was calculated [12].

### Gene set enrichment analyses

To access the biological functions and pathways, the Gene Set Enrichment Analysis (GSEA) software (https://www.gsea-msigdb.org/gsea/downloads.jsp) was used to perform pathway analysis [13]. The gene sets h.all.v7.4.symbols.gmt and c2.cp.kegg.v7.4.symbols.gmt were chosen as the reference gene set. The normalized enrichment score (|NES|> 1), nominal *p* value < 0.05 (NOM *p* value), and FDR adjusted *q*-value < 0.25 were considered as significant pathway enrichment. The top five significantly enriched signaling pathways were demonstrated.

### Implication of SYNGR2 expression in tumor immune microenvironment

The ESTIMATE algorithm was used to analysis the difference of stromal score, and immune score by the R package “estimate” [14]. The CIBERSORT algorithm was applied to assess the levels of 22 infiltrating immune cell subtypes [15, 16]. CIBERSORT can compute the abundance of specific cell types in a mixed sample based on the bulk expression. The R packages “limma” and “CIBERSORT” were used.

### Correlation analysis of SYNGR2 with TMB, MSI, checkpoint genes, and immunophenotype scores

We used the “maftools” package in R software to organize the single-nucleotide variants (SNV) data downloaded from the TCGA database in multiple alignment format. We also assessed tumor mutation burden (TMB) for each sample. Microsatellite instability (MSI) refers to the nucleotide insertions or deletions in the microsatellite loci. Spearman’s rank method was used to determine the correlation of SYNGR2 with TMB and MSI. The correlation results for TMB and MSI were visualized in radar maps. To explore the prognostic value of SYNGR2 on immunotherapy, we analyzed the relationship between SYNGR2 and 29 common immune checkpoint genes. The immunophenotype scores (IPS) downloaded from The Cancer Immunome Atlas (TCIA, https://tcia.at/home) were leveraged to predict the clinical response to immunotherapy in SYNGR2^high^ and SYNGR2^low^ expression groups [17].

### Establishment and evaluation of the nomogram

SYNGR2 expression and the pathologic T, N, M stage were used to build a nomogram, which is an effective and convenient approach for estimating the survival in individual patients [18]. The calibration curve was performed to verify the prediction accuracy of the nomogram.

### Statistical analysis

R version 4.1.3 was used for all statistical studies. The survival curve was plotted by K–M plotter. Wilcoxon test was used to compare the differences between two groups, and Spearman analysis was used to calculate the correlation coefficients. Double-tailed *p* < 0.050 was considered statistically significant.

## Result

### SYNGR2 expression analysis in human pan‑cancer

**Fig. 1:**
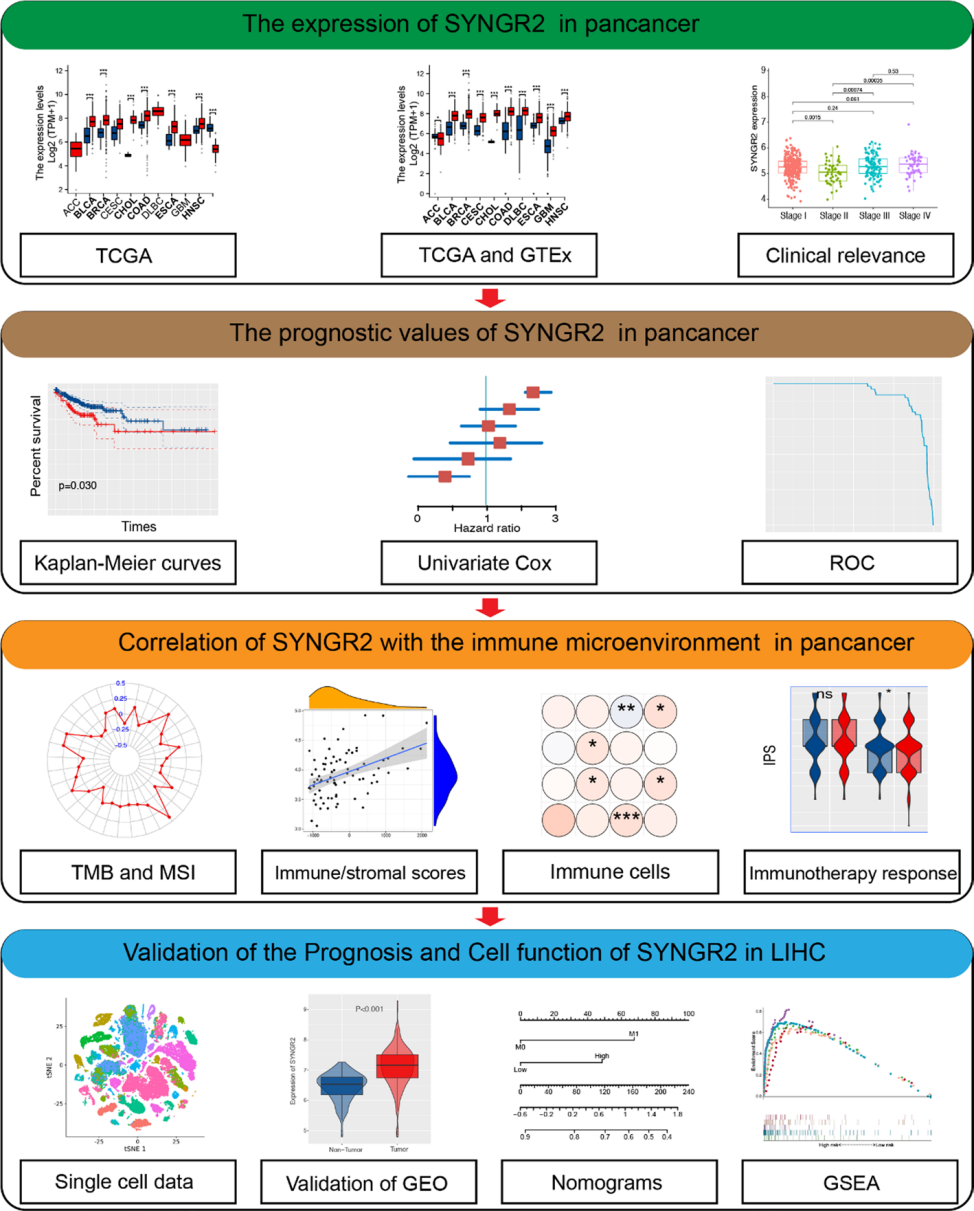
The flowchart for comprehensive analysis

**Fig. 2:**
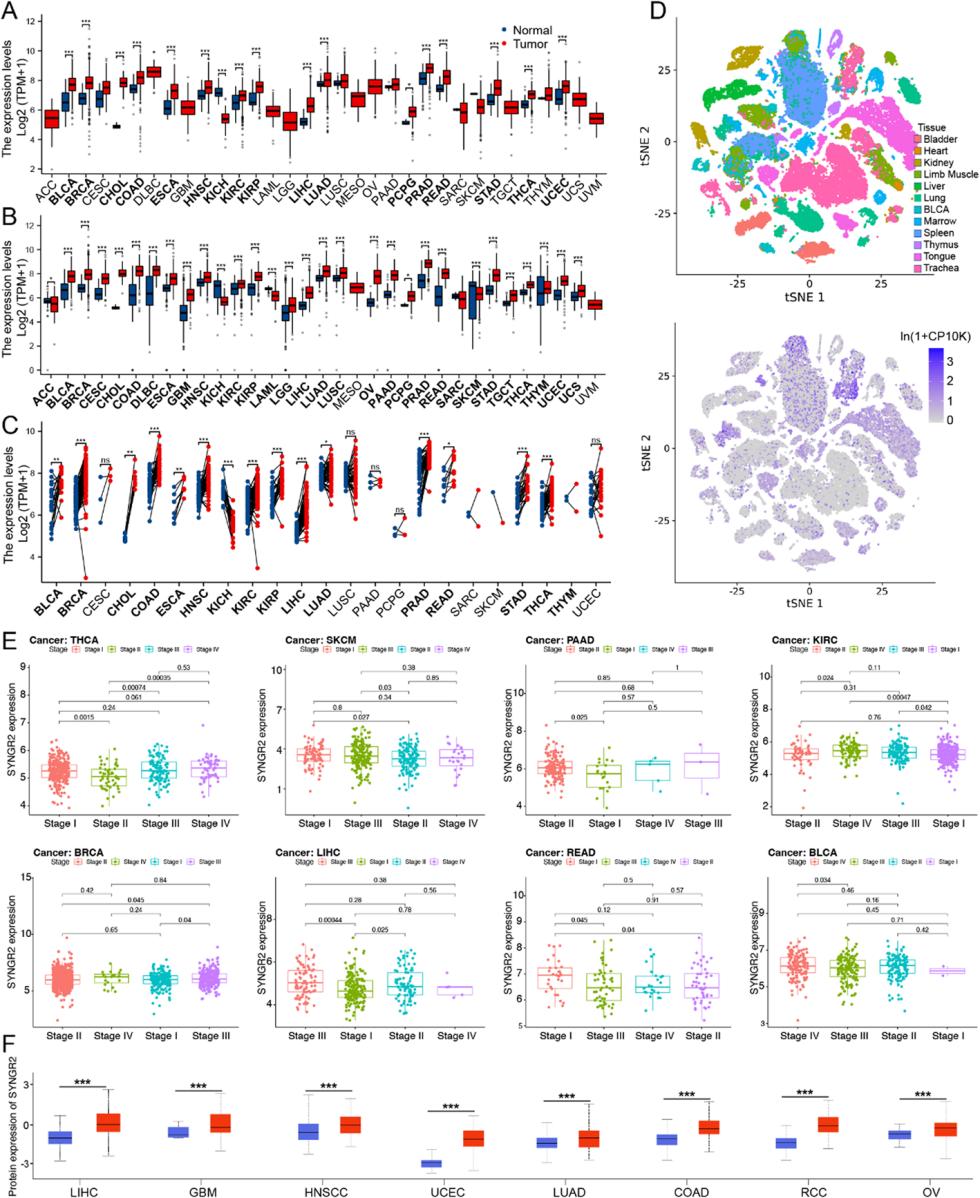
The expression of SYNGR2 in pan-cancer and different pathological stages. **A**. Differential expression of SYNGR2 in normal and tumor samples of 33 tumors in The Cancer Genome Atlas (TCGA) database. **B**. Data from TCGA and Genotype-Tissue Expression (GTEx) database showed differential expression of SYNGR2 in multiple cancers. **C**. Differential expression of SYNGR2 in cancers and normal tissues from TCGA dataset. **D**. T-SNE plot showing the expression of SYNGR2 in human tissue. **E**. Expression of SYNGR2 in different pathological stages of indicated tumors. **F**. SYNGR2 protein expression levels between tumor and respective normal tissues for 8 types of cancers. *p < 0.05, **p < 0.01, and ***p < 0.001

The flowchart of our study in Fig. 1. The RNA expression of SYNGR2 in pan-cancer data was first evaluated using the TCGA database. The results revealed that SYNGR2 mRNA level was significantly higher expressed in BLCA, BRCA, CHOL, LIHC, and other tumor tissues in comparison to corresponding normal tissues (Fig. 2A). Taking into account the lack of normal samples in the TCGA database for some cancer types, we integrated the GTEx database for further analysis and the results showed that the expression level of SYNGR2 in most tumor tissues are much higher than the corresponding control tissues (Fig. 2B). In paired samples, the expression of SYNGR2 in tumor tissues of BLCA, BRCA, CHOL, and others are significantly higher than the corresponding control tissues (Fig. 2C). Overall, SYNGR2 expression was significantly upregulated in various cancers. Next, human tissue were classified into 12 types as reported previously using the dimensional reduction method which was called t-distributed stochastic neighbor embedding (t-SNE), including bladder, heart, kidney, limb muscle, liver, lung, BLCA, marrow, spleen, thymus, tongue, trachea. The results suggested that SYNGR2 was highly expressed in liver, lung, marrow, spleen and tongue (Fig. 2D). Additionally, GEPIA-based analysis showed that SYNGR2 was differentially expressed in different stages in THCA, SKCM, PAAD, KIRC, BRCA, LIHC, READ, and BLCA (Fig. 2E). The UALCAN online tool confirmed that SYNGR2 protein levels were significantly upregulated in LIHC, GBM, HNSCC, UCEC, LUAD, COAD, RCC, and OV (Fig. 2F).

### Prognostic potential of SYNGR2 in pan-cancer

**Fig. 3:**
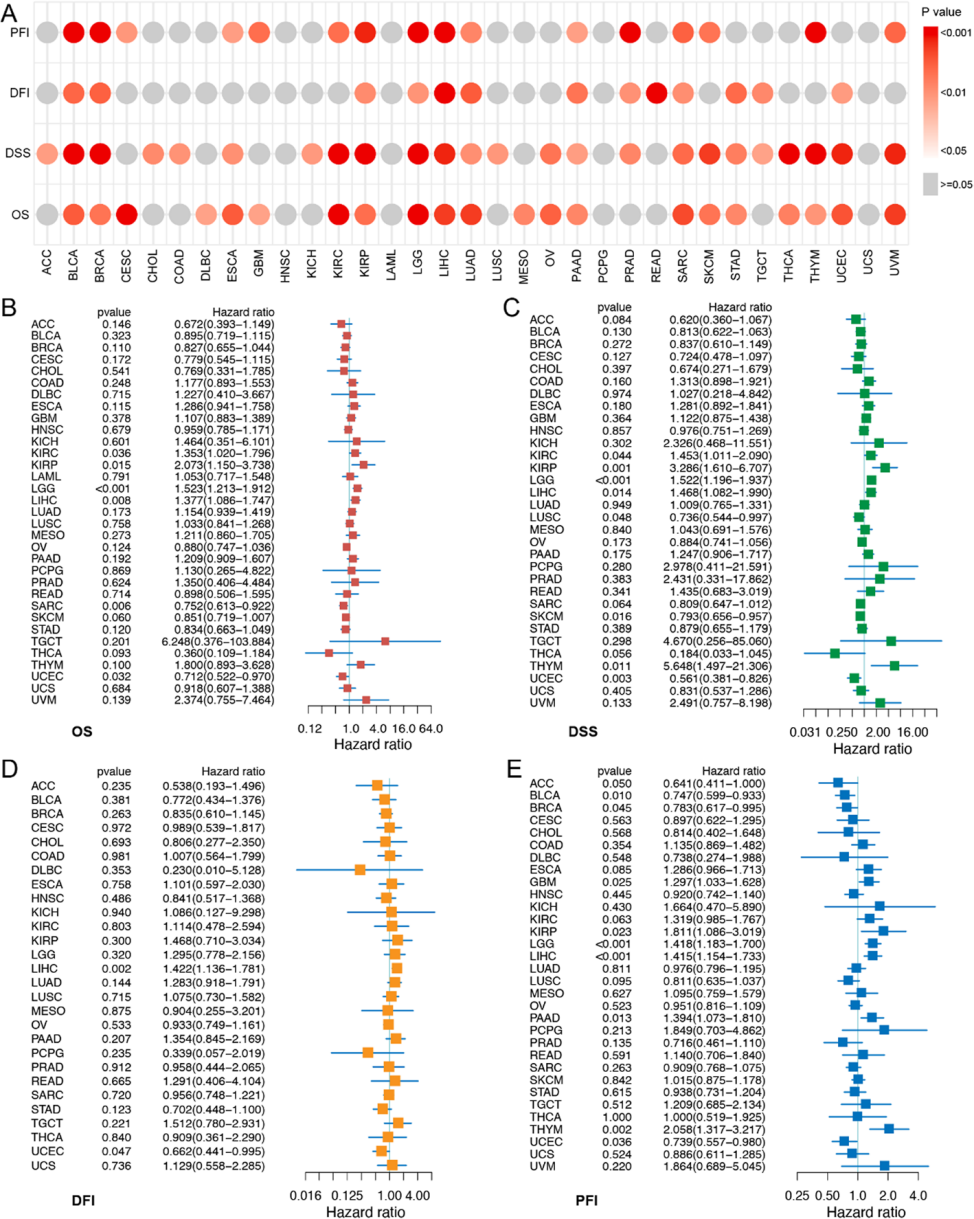
Correlation between SYNGR2 gene expression and prognosis in pan-cancer. **A**. Kaplan–Meier survival analyses were performed to determine the association between SYNGR2 and Overall Survival (OS), Disease-Specific Survival (DSS), Disease-Free Interval (DFI), Progression-Free Interval (PFI). **B**–**E**. The forest plots of univariate Cox regression analysis for OS (**B**), DSS (**C**), DFI (**D**), and PFI (**E**)

We investigated the clinical relevance of SYNGR2 in the different tumor types using Kaplan–Meier survival analysis (Fig. 3A; Additional file 1: Figs. S1–S4) regarding OS, DSS, DFI, and PFI. For example, the results showed that high SYNGR2 levels were associated with poor OS in BRCA, DLBC, GBM, MESO, PAAD, SKCM, STAD, THCA, and THYM (all *p* < 0.05), BLCA, ESCA, KIRP, LUAD, OV, SARC, and UCEC (all* p* < 0.01), CESC, KIRC, LGG, LIHC, and UVM (all *p* < 0.001). It is worth noting that SYNGR2 was most associated with the survival of LIHC (all *p* < 0.001). Univariate Cox regression analyses were further conducted to assess the associations of SYNGR2 with prognosis (Fig. 3B–E). Univariate Cox regression analysis showed that high expression of SYNGR2 significantly reduces the OS in 6 types of cancers including KIRC, KIRP, LGG, LIHC, SARC, and UCEC (Fig. 3B). Results for DSS, DFI, and PFI are shown in Fig. 3C–E. These results suggested that SYNGR2 expression had a powerful prognostic ability in different tumors.

### Diagnostic value of SYNGR2 for pan-cancer

**Fig. 4:**
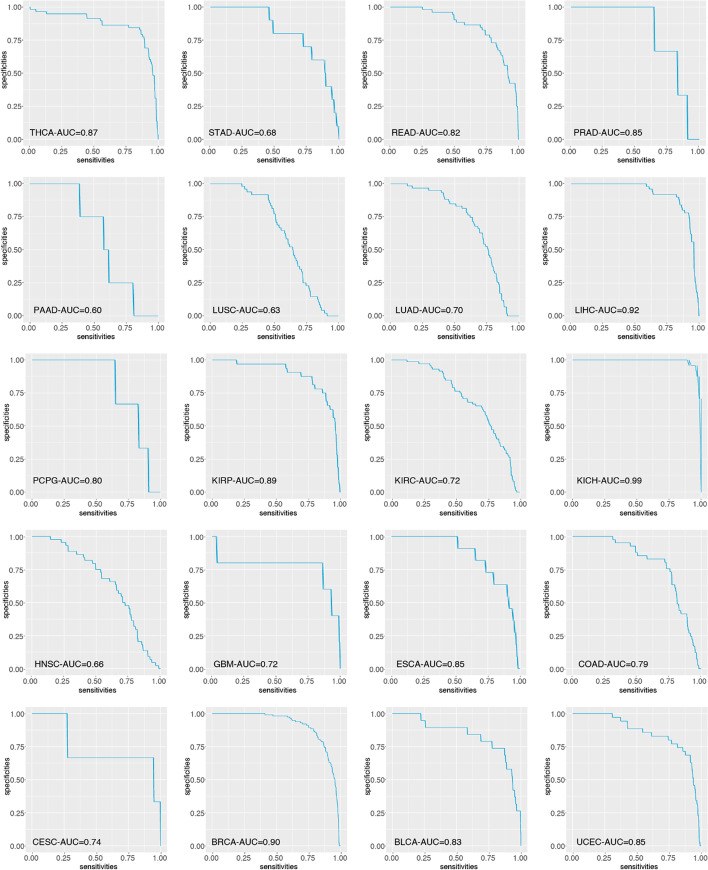
The ROC curve was used to assess the diagnostic value of SYNGR2 expression levels between tumor and normal tissues

Furtherly, we used receiver operating characteristic (ROC) curve to assess the diagnostic Value of SYNGR2 expression levels between tumor and normal tissues. The area under curve (AUC) values for ROC analysis in each cancer are shown in Fig. 4. The AUC values suggested that SYNGR2 could reliably distinguish tumor from normal tissues for various types of cancer, especially THCA (AUC = 0.87), PRAD (AUC = 0.85), LIHC (AUC = 0.92), KIRP (AUC = 0.89), KICH (AUC = 0.99), ESCA (AUC = 0.85), BRCA (AUC = 0.90), and UCEC (AUC = 0.85).

### Correlations of SYNGR2 expression with TMB, MSI, and immune score in pan-cancer

**Fig. 5:**
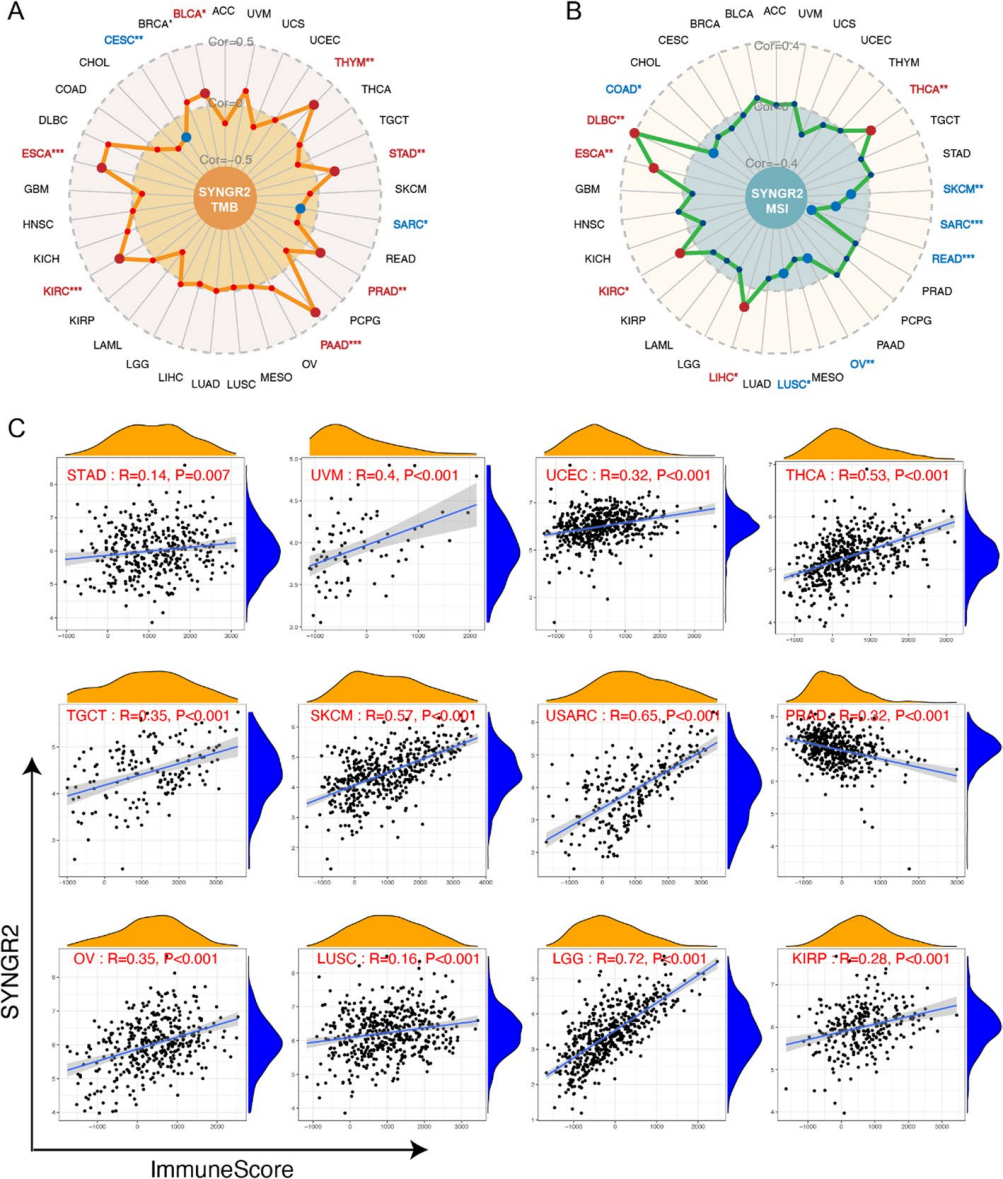
Correlation between the expression of SYNGR2 with TMB, MSI, and immune scores in pan-cancer. **A**–**B**. Radar maps of correlations between SYNGR2 expression and TMB (**A**) or MSI (**B**). **C**. The scatter plots of correlation between SYNGR2 expression and immune scores in multiple cancers. *p < 0.05, **p < 0.01, and ***p < 0.001

Both tumor mutation burden (TMB) and microsatellite instability (MSI) are pivotal characteristics of tumors. TMB, which counts the number of somatic mutations per megabase (mut/Mb), is an emerging potential biomarker for immunotherapy [19]. MSI refers to an abnormal DNA mismatch repair function where microsatellite replication errors are not corrected and accumulate, causing changes in microsatellite sequence length or base composition. MSI can be used as a prognostic marker for cancer patients [20]. Here, we evaluated the correlation of SYNGR2 expression with TMB and MSI (Fig. 5A–B). Results showed that SYNGR2 expression was positively related to TMB in BLCA, STAD, PRAD, and other tumor tissues; while negatively associated with TMB in SARC, and CESC. In terms of MSI, the SYNGR2 expression was positively related to MSI in DLBC, ESCA, KIRC, LIHC, and THCA; while negatively related to MSI in COAD, LUSC, OV, and other tumor tissues.

The tumor microenvironment (TME) plays an important role in the development of cancer. The TME consists of tumor cells, immune cells, stromal cells, etc. The Estimation of Stromal and Immune cells in Malignant Tumor tissues using Expression data (ESTIMATE) algorithm was used to evaluate infiltrating immune and stromal cells by calculating immune scores and stromal scores. SYNGR2 expression was found to positively correlate with the immune score in STAD, UVM, UCEC, and other tumor tissues, but negatively correlated with the immune score in PRAD (Fig. 5C). Regarding stromal score, a positive correlation with SYNGR2 expression was seen in GBM, KICH, SARC, SKCM, TGCT, THCA, UVM, and a negative correlation in BRCA, COAD, PRAD, and PAAD (Additional file 1: Fig. S5).

### Correlation between SYNGR2 and immune cell infiltration, immunophenotype scores in different tumors

**Fig. 6:**
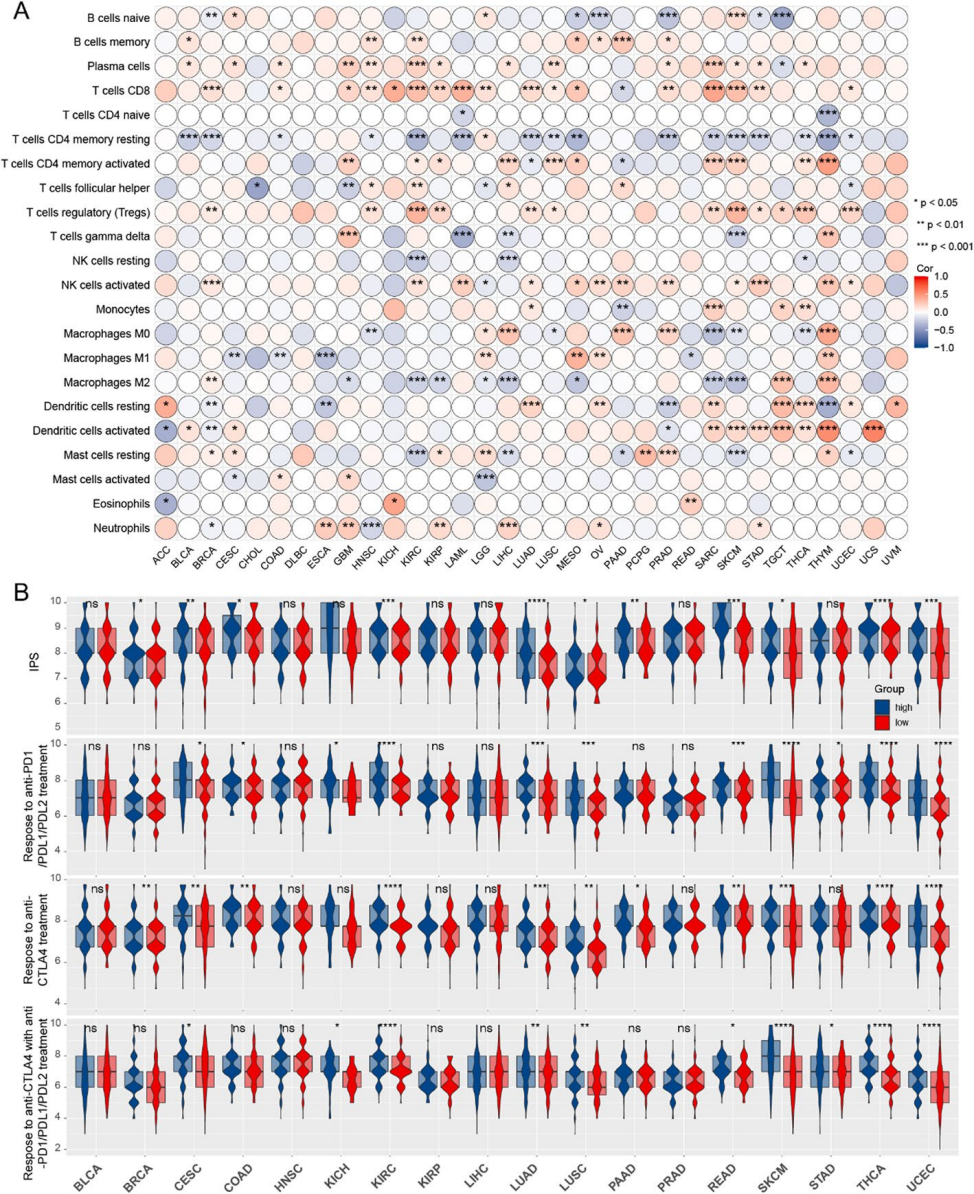
Correlation between the expression of SYNGR2 and immune infiltration in pan-cancer. **A**. The abundance of 22 immune cells calculated by CIBERSORT. **B**. The difference of IPS between SYNGR2^high^ and SYNGR2^low^ expression groups. *p < 0.05, **p < 0.01, and ***p < 0.001

Previous studies have found that the expression of SYNGR2 in esophageal cancer is related to immune cell infiltration [7]. Based on this, the Estimating Relative Subsets of RNA Transcripts (CIBERSORT) algorithm was used to determine the composition of 22 immune cell subsets based on gene expression profiles. Results indicated that SYNGR2 was significantly associated with immune cell subsets in BRCA, GBM, HNSC, and other tumor tissues. SYNGR2 exhibited positive associations with Plasma cells, T regulatory cells (Tregs), CD8^+^ T cells, activated memory CD4^+^ T cells, activated NK cells, activated Dendritic cells, and negative associations with native B cells, resting memory CD4^+^ T cells, M2 Macrophages in the majority of tumors (Fig. 6A).

As presented in Fig. 6B, we determined the sensitivity to immune checkpoint inhibitors for the different tumors. Our results showed that the SYNGR2^high^ group possessed a significantly higher immunophenotype scores (IPS) than the SYNGR2^low^ group in BRCA, CESC, COAD, KIRC, and other tumor tissues. Moreover, the SYNGR2^high^ group was more likely to gain benefits from anti-PD1/PDL1/PDL2 therapies in CESC, COAD, KICH, other tumor tissues, and respond to anti-cytotoxic T-lymphocyte associated protein 4 (CTLA-4) therapy in BRCA, CESC, COAD, and other tumor tissues. Finally, the SYNGR2^high^ group was prone to respond to the combination of the two immunotherapies in CESC, KICH, KIRC, and other tumor tissues.

### Correlations of SYNGR2 expression with HLA, immune checkpoint genes, and chemokines in LIHC

In the above analysis, we discovered a correlation between SYNGR2 expression and prognosis of multiple tumors and immune cell infiltration. We found that SYNGR2 was significantly associated with LIHC, especially in terms of prognosis and diagnostic value. Therefore, to further understand the role of SYNGR2 in LIHC, we analyzed the expression of human leukocyte antigen (HLA), immune checkpoint genes, and chemokines.

**Fig. 7:**
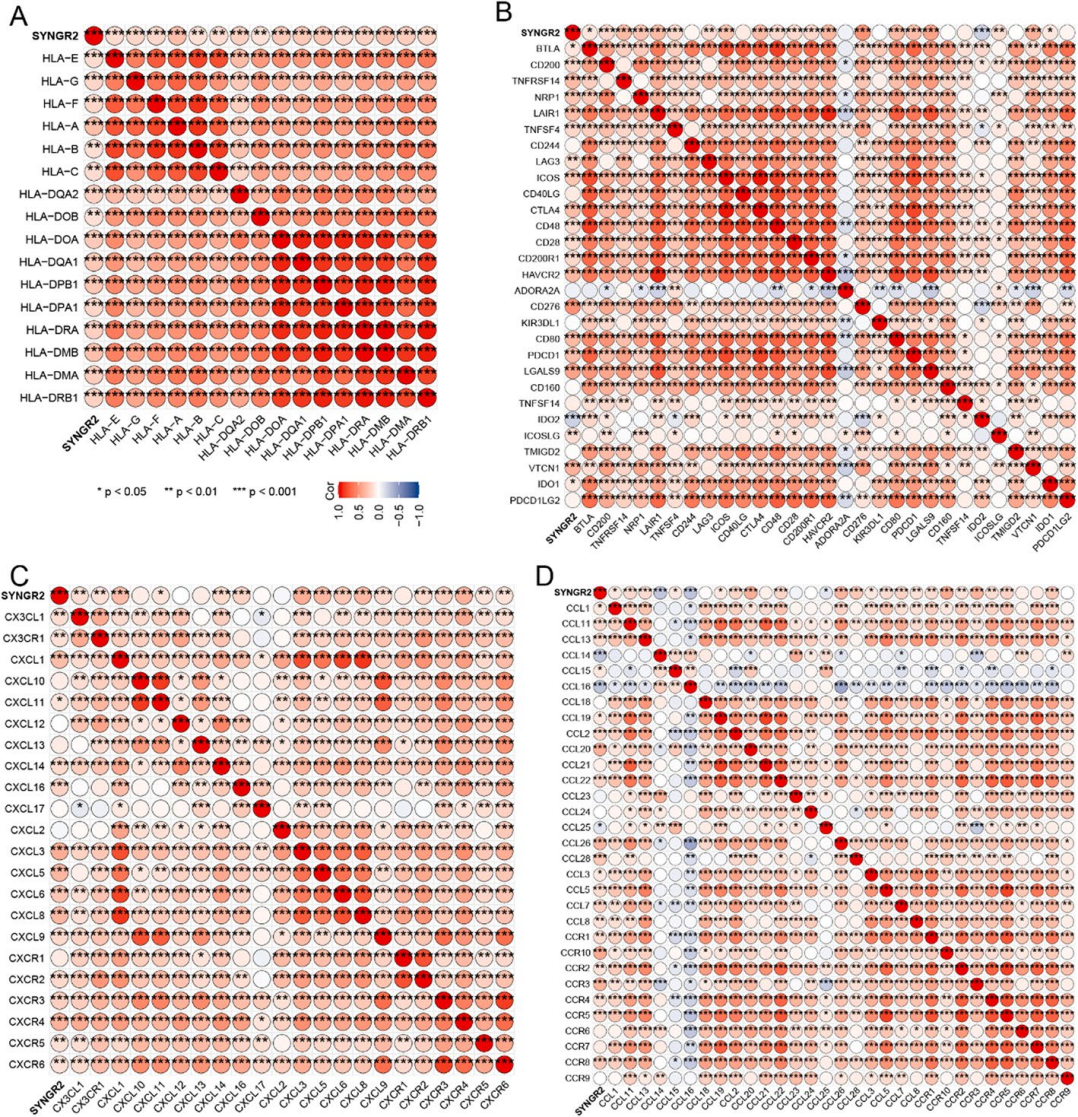
Assessment of immunotherapy response in LIHC. **A**. Correlations of SYNGR2 Expression with HLA gene family. **B**. Correlation of SYNGR2 Expression with immune checkpoint genes. **C**, **D**. Correlation between SYNGR2 and chemokines in tumors. *p < 0.05, **p < 0.01, and ***p < 0.001

Class I HLA is the expression product of the human major histocompatibility complex (MHC) and is located on the short arm of chromosome 6 [21]. The deficiency of HLA may impair cells’ ability to present neoantigens and cause immune tolerance [22]. The results showed that SYNGR2 was positively correlated with all HLA members (Fig. 7A). Cancer immunotherapy represented by immune checkpoint inhibitors (ICIs) offers a promising treatment option for LIHC [23]. To further investigate the predictive role of SYNGR2 Expression in immunotherapy, we examined the correlation between SYNGR2 and immune checkpoint (ICP) genes, (Fig. 7B). Many ICP genes were positively correlated with SYNGR2, especially in CD200, CD200R1, CTLA4, LAIR1, ICOS, CD276, PDCD1, CDD80, and VTCN1. Chemokines and chemokine receptors play an important role in the development and progression of LIHC. Different chemokines and receptors have different roles in hepatocellular carcinoma [24]. Chemokines can be classified into 4 categories according to the position of the two conserved N-terminal cysteine residues: CC, CXC, C and CX3C [25]. Chemokines and chemokine receptors mediate the movement of immune cells in the TME [26]. We found that SYNGR2 was highly positive correlated with CXCL1, CXCL3, CXCL5, CXCL8, CXCR4, CCL2, CCL20, CCL26, CCL28, CCL7, CCR10, CCR3, and CCR8 expression in LIHC samples (Fig. 7C–D).

### The expression, prognostic value and signaling pathway of SYNGR2 in LIHC

**Fig. 8:**
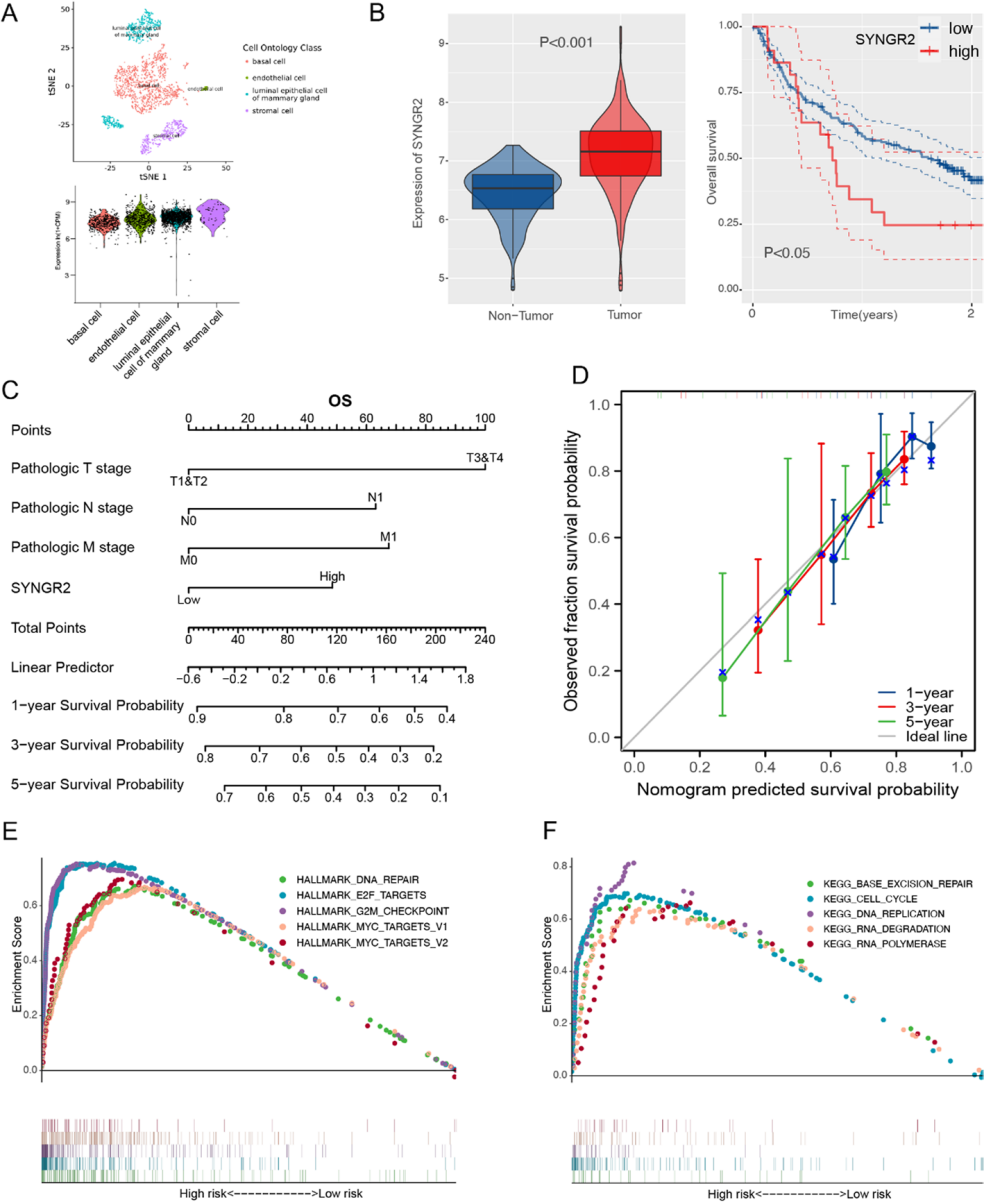
GSEA regarding SYNGR2 for LIHC and construction of a SYNGR2-based prognostic prediction model. **A**. T-SNE plot showing the expression of SYNGR2 in Cell Ontology Class. **B**. The SYNGR2 expression and prognosis analysis in GSE14520 dataset. **C**. Nomogram for predicting the proportion of patients with OS. **D**. Calibration curves of the nomogram for 1, 3 and 5 years. **E**, **F**. Enrichment results of HALLMARK and KEGG signaling pathways

First, we use the t-SNE method to classify Cell Ontology Class into 4 types based on the expression level of SYNGR2, including basal cell, endothelial cell, luminal epithelial cell of mammary gland, stromal cell. The results suggested that SYNGR2 was highly expressed in stromal cell (Fig. 8A). Additionally, results above were verified in GSE14520 dataset to further demonstrate the expression and prognosis of SYNGR2 in LIHC. The expression of SYNGR2 in tumor tissue is higher than that in non-tumor tissue (*p* < 0.001), and the SYNGR2^high^ group had a worse OS compared with the SYNGR2^low^ group in K-M curve (*p* < 0.05; Fig. 8B).

In order to investigate the application of SYNGR2 in cancer prognosis, we built a nomogram for predicting the OS, DSS, PFI of LIHC patients (Fig. 8C, Additional file 1: Fig. S6A, C). The pathologic T, N, M stage and SYNGR2 were included as prognostic factors in the nomogram. The calibration curve showed that the nomogram had a good ability in predicting possibility of 1-, 3-, 5-years OS, DSS, and PFI in LIHC (Fig. 8D, Additional file 1: Fig. S6B, D). These results demonstrated that the nomogram combining SYNGR2 expression and pathologic T, N, M stage had better predictive power for the OS, DSS, and PFI of LIHC patients, which might contribute to assess the prognosis of patients.

To explore the potential function of SYNGR2 in LIHC, we carried out a gene set enrichment analysis (GSEA). The top five signaling pathways significantly associated with SYNGR2 are exhibited in Fig. 8E–F. SYNGR2 was associated with DNA repair, E2F targets, MYC targets, cell cycle, DNA replication, and RNA polymerase. These results suggest that SYNGR2 plays a vital role in tumorigenesis and progression.

## Discussion

SYNGR2 belongs to the synaptogyrin gene family. The function of synaptogyrin is related to the release of neurotransmitters and the early neurons development [27]. The SYNGR2 gene is expressed at high levels in all tissues, except brain, and it plays an important role in biological processes such as cell exocytosis, the storage and transport of GLUT4 in the cytoplasmic membrane, and the formation and maturation of microvesicles in neuronal cells [5]. To date, the evidence of SYNGR2’s implications in cancer is very preliminary and lacks experimental validation. Li et al. [7] reported the elevated expression of SYNGR2 in ESCC. High SYNGR2 expression was associated with poorer OS in ESCC. SYNGR2 has an important reference value for the diagnosis of ESCC. Collectively, SYNGR2’s potential roles in carcinogenesis and cancer development are worthwhile to be further disclosed.

In the present study, we explored the pan-cancer expression profiles of SYNGR2, and the correlation between SYNGR2 aberrant expression and patient prognosis in different cancers. A pan-cancer analysis revealed that compared to normal tissues, significantly elevated mRNA expression levels of SYNGR2 were observed in 26 cancers, including BLCA, BRCA, CESC, and other tumor tissues. Furthermore, SYNGR2 expression levels increased with tumor progression in THCA, SKCM, PAAD, and other tumor tissues. In terms of prognosis, Kaplan–Meier survival and univariate Cox regression analysis demonstrated that patients with SYNGR2^high^ tumors were more likely to suffer inferior survival in BRCA, PAAD, BLCA, and other tumor tissues.

Cancer is a complicated disease involving complex reciprocal networks between tumor cells and the immune system. The tumor microenvironment (TME) is the internal environment in which the tumor cells are located, which includes not only the tumor cells themselves, but also the stromal cells, immune cells and other components [28]. The TME plays an important role in the proliferation, invasion and metastasis of cancer [29]. On the one hand, immune cells are primarily responsible for the elimination of tumor cells. The infiltration density and activity of immune cells have been demonstrated to be not only predictive of the response to ICBs [30], but also independent prognostic markers for tumor patients [31, 32]. On the other hand, tumor cells can recruit immunosuppressive cells to evade immune killing by altering their immunogenicity and biological properties [33]. We adopted ESTIMATE algorithm to determine stromal and immune scores for samples across 33 cancer types. Our exploration demonstrated that SYNGR2 is positively correlated with immune scores in most cancer types. However, the association of SYNGR2 with stromal scores varied among different tumors. This suggests that the role of SYNGR2 is tumor-specific. We also used the CIBERSORT algorithm to estimate the proportion of 22 immune cell subsets in each tumor sample. The correlation test indicated that SYNGR2 was extensively correlated to immune cell infiltrates in BRCA, GBM, HNSC, and other tumor tissues. In addition, we also found that aberrant SYNGR2 expression was correlated with increased immune cell infiltration of T regulatory cells (Tregs) in the majority of cancers. Tregs is an immunosuppressive cell that is abundant in the TME. Tregs inhibits the T cells activation and proliferation through various mechanisms, such as inhibition of MHC molecules and co-stimulatory molecules (CD80 and CD86) on the surface of antigen-presenting cells (APCs) to inhibit APC maturation and thus attenuate the interaction between APCs and T cells [34]. This further suggests that SYNGR2 may play an important regulatory role in the TME.

Given the correlation between SYNGR2 and TME, we further explored whether SYNGR2 is related to the response to immunotherapy. Tumor mutation burden (TMB) and microsatellite instability (MSI) have been described as powerful predictors of tumor behavior and response to immunotherapy [19, 20]. In this study, we evaluated the correlation of SYNGR2 expression with TMB and MSI. Our pan-cancer analysis revealed that SYNGR2 expression was positively related to TMB in BLCA, STAD, PRAD, and other tumor tissues. In terms of MSI, the SYNGR2 expression was positively related to MSI in DLBC, ESCA, KIRC, LIHC, and THCA. Next, in the study for IPS it was found that SYNGR2^high^ tumors exhibited a significantly higher IPS than the SYNGR2^low^ tumors. The IPS is the most comprehensive estimator of tumor immunogenicity. The IPS has shown remarkable performance in terms of predicting response to immunotherapies blocking CTLA and PD1 [17]. Overall, these results indicated that SYNGR2 might be related to the response to immunotherapy.

In this study, we observed the most robust relationship of aberrant SYNGR2 expression with patient prognosis in LIHC. Therefore, we validated the role of SYNGR2 in LIHC by constructing a prognostic risk score model, as well as evaluating the relationship between SYNGR2 and immune checkpoints. The nomogram including SYNGR2 and pathologic T, N, M stage showed good prognostic predictive performance. To further clarify the role of SYNGR2 in tumorigenesis and progression, we conducted a gene set enrichment analysis. The results suggested SYNGR2 was associated with DNA repair, E2F targets, MYC targets, cell cycle, DNA replication, and RNA polymerase. E2F, an important regulator of the cell cycle and apoptosis, plays an important role in the development of cancer [35]. Chen et al. [36] found that overexpression of E2F1 accelerated the proliferation of hepatocellular carcinoma cells and promoted tumor formation in vitro hepatocellular carcinoma cell lines and mouse models. MYC is one of the most widely investigated cancer-causing genes, being implicated in the formation, maintenance and progression of several different cancer types [37, 38]. Moreover, many ICP genes were positively correlated with SYNGR2 in LIHC. ICIs have caused a revolution in cancer care by reversing the immunosuppressive tumor microenvironment. In our research, CD200, CD200R1, CTLA4, LAIR1, ICOS, CD276, PDCD1, CDD80, and VTCN1 were correlated to SYNGR2. Notably, CTLA4 and PDCD1 /PD1 have received considerable attention as a putative immune checkpoint in TME. To sum up, the above analysis demonstrated that SYNGR2 played a tumor-promoting role and related to the response to ICP therapies in LIHC.

Although our pan-cancer analysis exhibited great performance, there were some limitations in our research. First, our findings should be explained cautiously due to the retrospective nature of the study. Second, all data for the article were obtained from public databases. Therefore, more clinical data are needed to validate the above results. Finally, the mechanism of SYNGR2 in LIHC prognosis remains unknown, a more in-depth investigation will be undertaken in vivo or in vitro.

In conclusion, SYNGR2 can be considered as a critical oncogene. Moreover, SYNGR2 is differentially expressed in a variety of tumors and aberrant expression is associated with the progression of the tumor, especially in LIHC. The aberrant SYNGR2 expression is associated with immune cell infiltration, immune scores, ICP genes, IPS, TMB, and MSI. Our findings demonstrated that SYNGR2 could be a potential biomarker, as well as a predictor of survival and immunotherapy in cancer treatment.

## Supplementary Information

**Additional file 1**: **Fig. S1**. Kaplan–Meier survival analysis for the association between the expression of SYNGR2 and overall survivalof pan-cancer. **Fig. S2**. Kaplan–Meier survival analysis for the association between the expression of SYNGR2 and disease-specific survivalof pan-cancer. **Fig. S3**. Kaplan–Meier survival analysis for the association between the expression of SYNGR2 and disease-free intervalof pan-cancer. **Fig. S4**. Kaplan–Meier survival analysis for the association between the expression of SYNGR2 and progression-free intervalof pan-cancer. **Fig. S5**. Correlation between the expression of SYNGR2 and stromal scores in pan-cancer. **Fig. S6**. Prediction of DSS and PFI by SYNGR2-based prognostic models. (A, C). Nomogram for predicting the proportion of patients with DSS, and PFI. (B, D). Calibration curves of the nomogram for 1, 3, and 5 years.

## Data Availability

The original contributions presented in the study are included in the article/Additional file. Further inquiries can be directed to the corresponding authors.

## Abbreviations

SYNGR2: Synaptogyrin-2
TCGA: The cancer genome atlas
GTEx: Genotype-tissue expression
GEPIA: Gene expression profiling interactive analysis
TCIA: The cancer immunome atlas
GSEA: Gene set enrichment analysis
ACC: Adrenocortical carcinoma
BLCA: Bladder urothelial carcinoma
BRCA: Breast invasive carcinoma
CESC: Cervical squamous cell carcinoma and endocervical adenocarcinoma
CHOL: Cholangiocarcinoma
COAD: Colon adenocarcinoma
DLBC: Lymphoid neoplasm diffuse large b-cell lymphoma
ESCA: Esophageal carcinoma
GBM: Glioblastoma multiforme
HNSC: Head and neck squamous cell carcinoma
KICH: Kidney chromophobe
KIRC: Kidney renal clear cell carcinoma
KIRP: Kidney renal papillary cell carcinoma
LAML: Acute myeloid leukemia
LGG: Brain lower grade glioma
LIHC: Liver hepatocellular carcinoma
LUAD: Lung adenocarcinoma
LUSC: Lung squamous cell carcinoma
MESO: Mesothelioma
OV: Ovarian serous cystadenocarcinoma
PAAD: Pancreatic adenocarcinoma
PCPG: Pheochromocytoma and paraganglioma
PRAD: Prostate adenocarcinoma
READ: Rectum adenocarcinoma
SARC: Sarcoma
SKCM: Skin cutaneous melanoma
STAD: Stomach adenocarcinoma
TGCT: Testicular germ cell tumors
THYM: Thymoma
THCA: Thyroid carcinoma
UCS: Uterine carcinosarcoma
UCEC: Uterine corpus endometrial carcinoma
UVM: Uveal melanoma
OS: Overall survival
DSS: Disease-specific survival
DFI: Disease-free interval
PFI: Progression free interval
IPS: Immunophenotype scores
TMB: Tumor mutation burden
TME: The tumor microenvironment
MSI: Microsatellite instability
ICP: Immune checkpoints
ROC: Receiver operating characteristic
ICIs: Immune checkpoint inhibitors
T-SNE: T-distributed stochastic neighbor embedding
SNV: Single-nucleotide variants
AUC: Area under curve
HR: Hazard ratio
CI: Confidence interval
APCs: Antigen-presenting cells
Tregs: T regulatory cells
HLA: Human leukocyte antigen
MHC: Major histocompatibility complex
CTLA-4: Anti-cytotoxic T-lymphocyte associated protein 4
