# Supplementary material for: Pan-cancer analysis of SYNGR2 with a focus on clinical implications and immune landscape in liver hepatocellular carcinoma
Source: BMC Bioinformatics. 2023 May 11;24:192. doi: 10.1186/s12859-023-05323-y (PMC10173524; doi:10.1186/s12859-023-05323-y)
Supplement: Supplementary file 1 — Additional file 1: Fig. S1. Kaplan–Meier survival analysis for the association between the expression of SYNGR2 and overall survivalof pan-cancer. Fig. S2. Kaplan–Meier survival analysis for the association between the expression of SYNGR2 and disease-specific survivalof pan-cancer. Fig. S3. Kaplan–Meier survival analysis for the association between the expression of SYNGR2 and disease-free intervalof pan-cancer. Fig. S4. Kaplan–Meier survival analysis for the association between the expression of SYNGR2 and progression-free intervalof pan-cancer. Fig. S5. Correlation between the expression of SYNGR2 and stromal scores in pan-cancer. Fig. S6. Prediction of DSS and PFI by SYNGR2-based prognostic models. (A, C). Nomogram for predicting the proportion of patients with DSS, and PFI. (B, D). Calibration curves of the nomogram for 1, 3, and 5 years. [file 12859_2023_5323_MOESM1_ESM.docx]

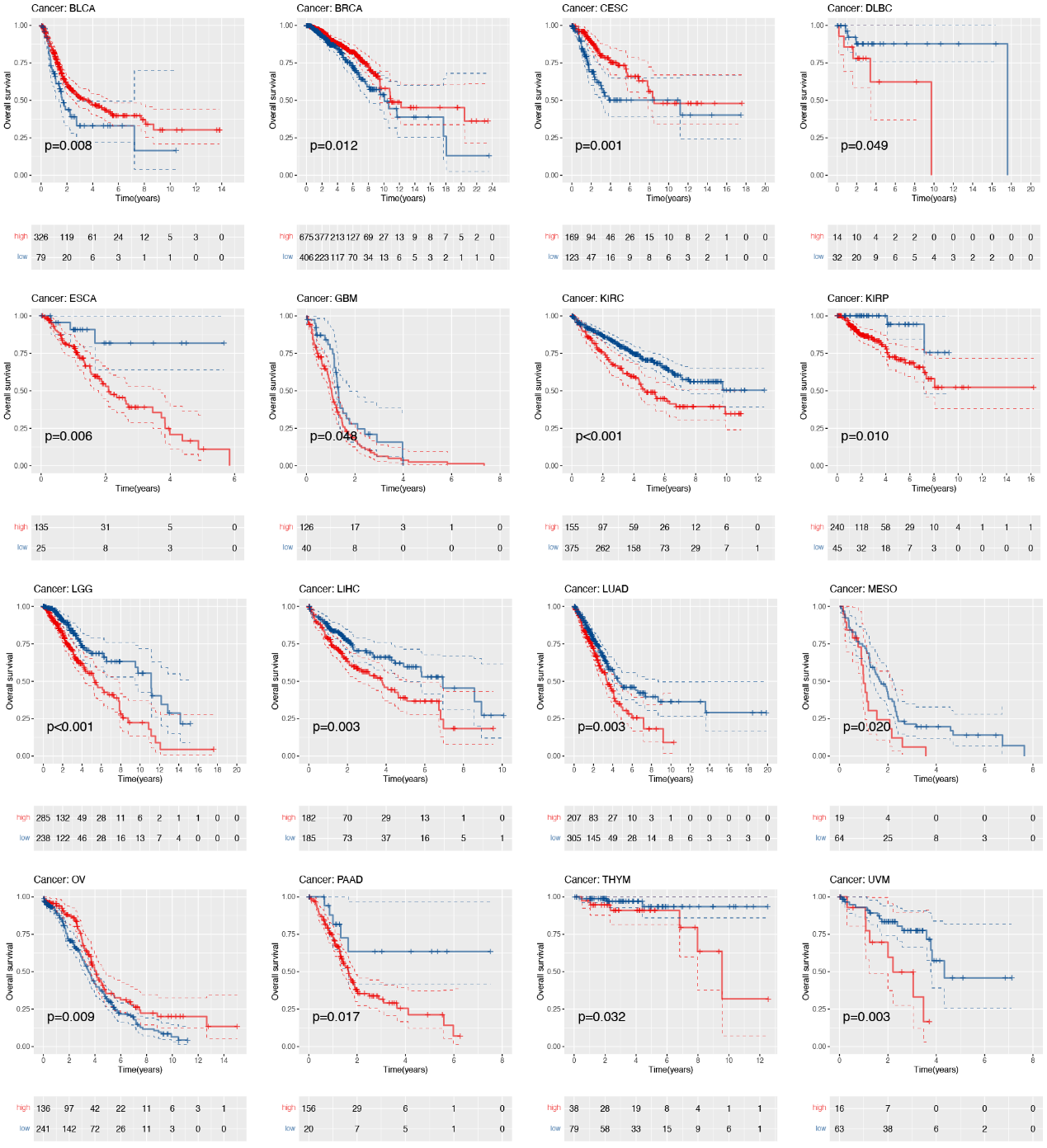


**Supplementary figure 1.** Kaplan-Meier survival analysis for the association between the expression of SYNGR2 and overall survival (OS) of pan-cancer.


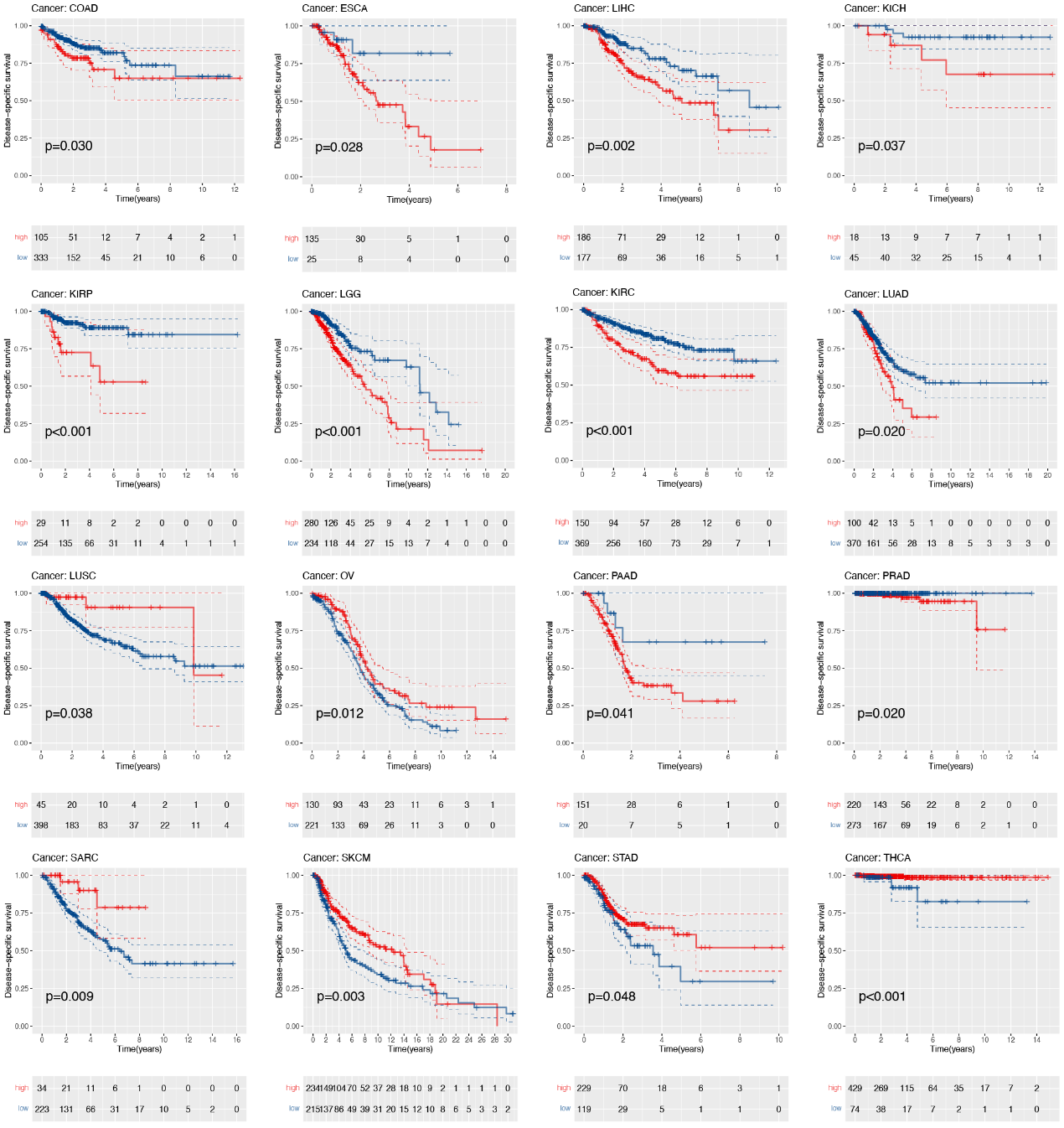


**Supplementary figure 2.** Kaplan-Meier survival analysis for the association between the expression of SYNGR2 and disease-specific survival (DSS) of pan-cancer.


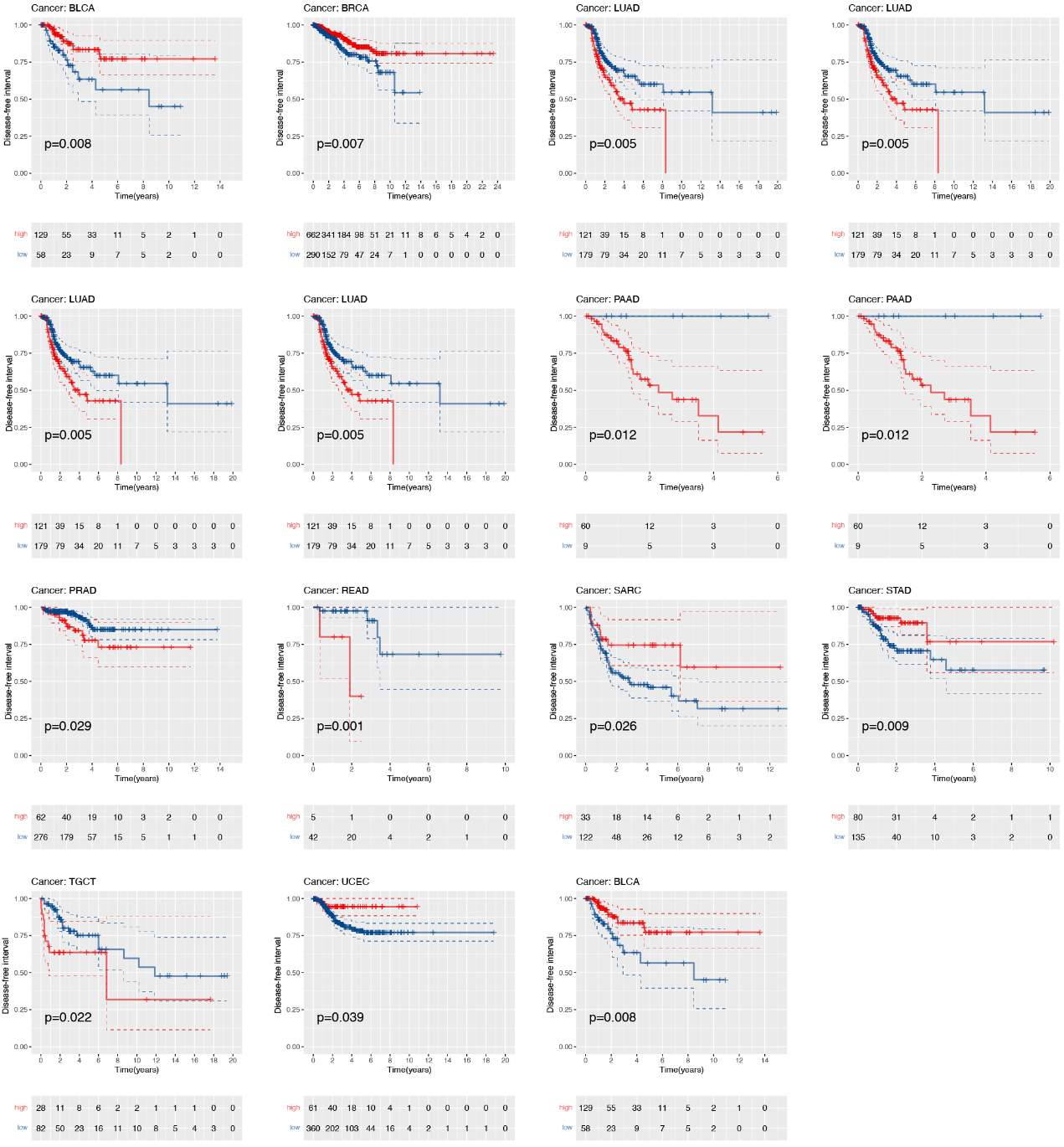


**Supplementary figure 3.** Kaplan-Meier survival analysis for the association between the expression of SYNGR2 and disease-free interval (DFI) of pan-cancer.


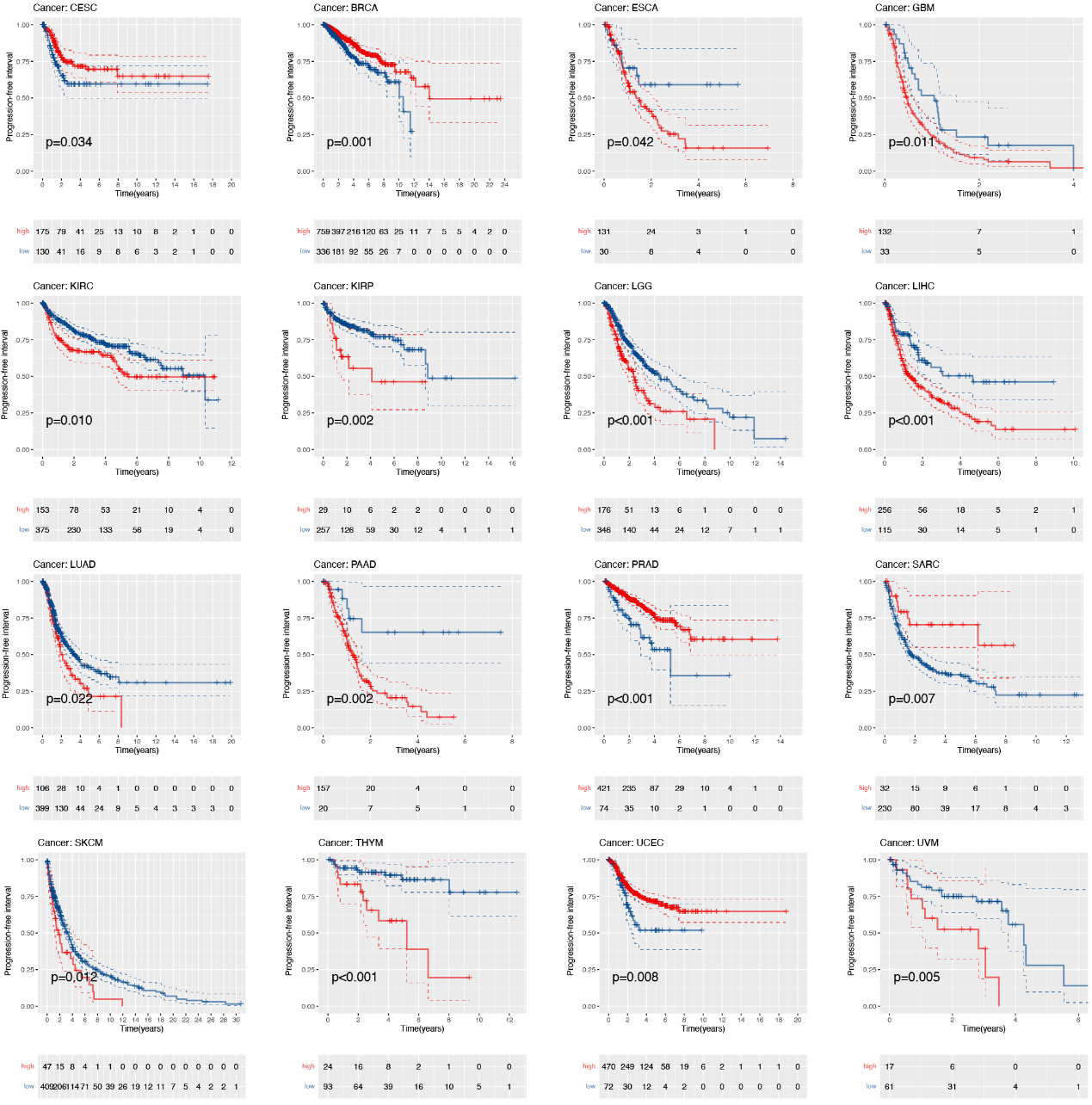


**Supplementary figure 4.** Kaplan-Meier survival analysis for the association between the expression of SYNGR2 and progression-free interval (PFI) of pan-cancer.


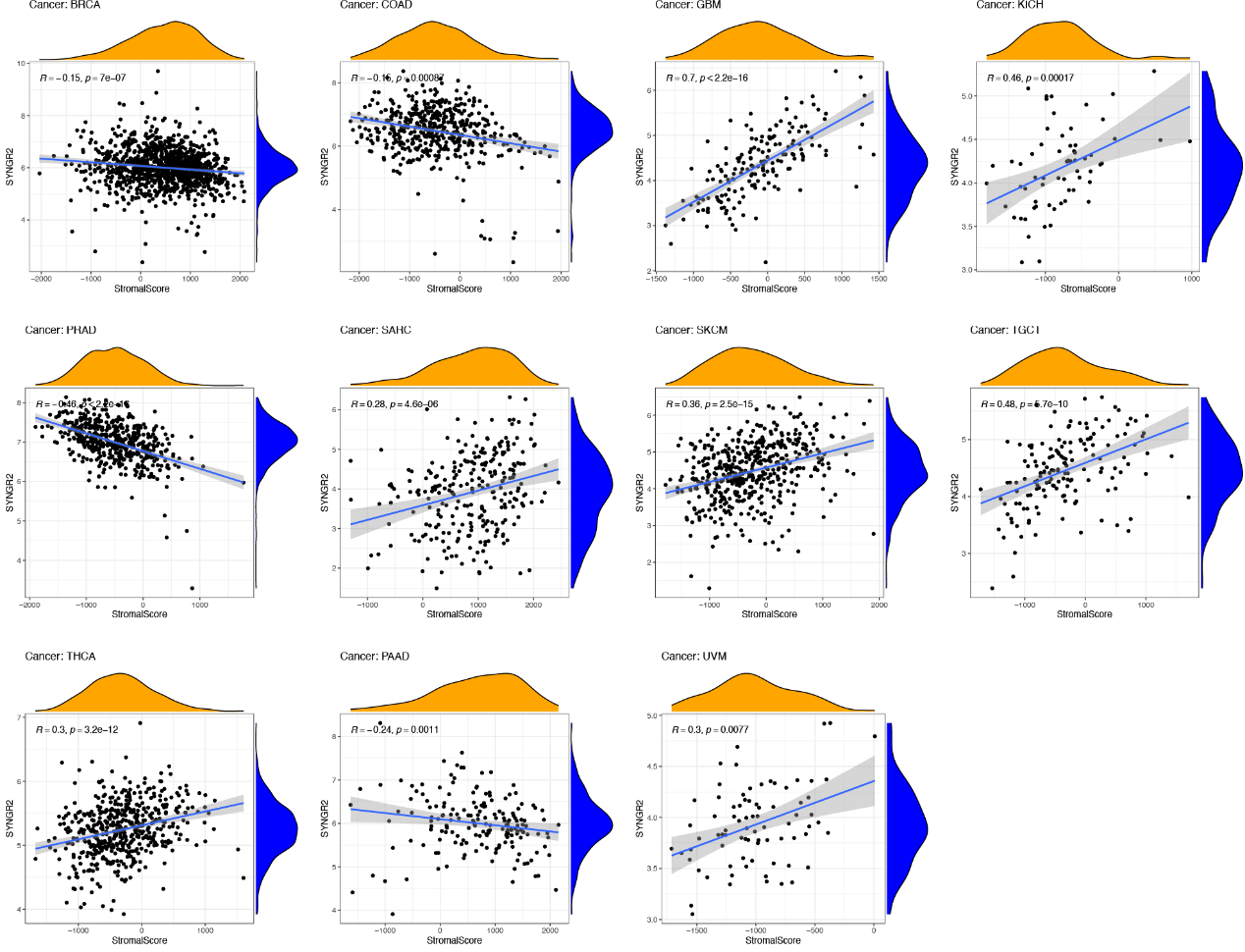


**Supplementary figure 5.** Correlation between the expression of SYNGR2 and stromal scores in pan-cancer.


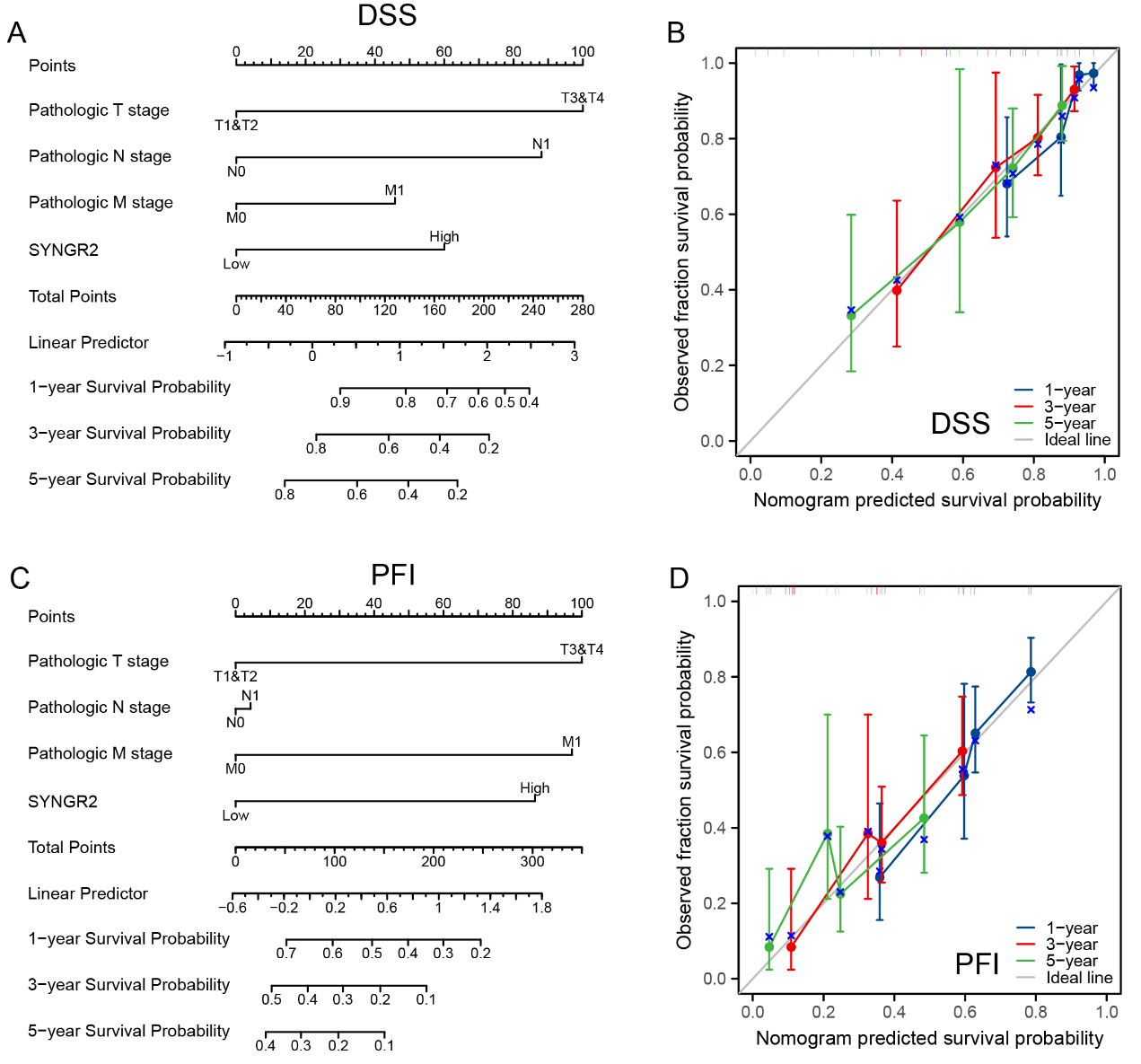


**Supplementary figure 6.** (A, C). Nomogram for predicting the proportion of patients with DSS, and PFI. (B, D). Calibration curves of the nomogram for 1, 3, and 5 years.
